# Weighted Co-Expression Network Analysis Identifies RNF181 as a Causal Gene of Coronary Artery Disease

**DOI:** 10.3389/fgene.2021.818813

**Published:** 2022-02-10

**Authors:** Ruoyu Dang, Bojian Qu, Kaimin Guo, Shuiping Zhou, He Sun, Wenjia Wang, Jihong Han, Ke Feng, Jianping Lin, Yunhui Hu

**Affiliations:** ^1^ State Key Laboratory of Medicinal Chemical Biology, College of Pharmacy and Tianjin Key Laboratory of Molecular Drug Research, Nankai University, Tianjin, China; ^2^ Pharmaceutical Intelligence Platform, Tianjin International Joint Academy of Biomedicine, Tianjin, China; ^3^ GeneNet Pharmaceuticals Co. Ltd., Tianjin, China; ^4^ The State Key Laboratory of Core Technology in Innovative Chinese Medicine, Tasly Academy, Tasly Holding Group Co., Ltd, Tianjin, China; ^5^ College of Life Sciences, State Key Laboratory of Medicinal Chemical Biology, Key Laboratory of Bioactive Materials of Ministry of Education, Nankai University, Tianjin, China

**Keywords:** WGCNA (weighted gene co-expression network analyses), coronary artery disease, heart tissue, GWAS meta-analysis, diagnostic biomarkers

## Abstract

**Background:** Coronary artery disease (CAD) exerts a global challenge to public health. Genetic heritability is one of the most vital contributing factors in the pathophysiology of CAD. Co-expression network analysis is an applicable and robust method for the interpretation of biological interaction from microarray data. Previous CAD studies have focused on peripheral blood samples since the processes of CAD may vary from tissue to blood. It is therefore necessary to find biomarkers for CAD in heart tissues; their association also requires further illustration.

**Materials and Methods:** To filter for causal genes, an analysis of microarray expression profiles, GSE12504 and GSE22253, was performed with weighted gene co-expression network analysis (WGCNA). Co-expression modules were constructed after batch effect removal and data normalization. The results showed that 7 co-expression modules with 8,525 genes and 1,210 differentially expressed genes (DEGs) were identified. Furthermore, Gene Ontology (GO) and Kyoto Encyclopedia of Genes and Genomes (KEGG) enrichment analyses were conducted. Four major pathways in CAD tissue and hub genes were addressed in the Hybrid Mouse Diversity Panel (HMDP) and Human Protein Atlas (HPA), and isoproterenol (ISO)/doxycycline (DOX)-induced heart toxicity models were used to validate the hub genes. Lastly, the hub genes and risk variants were verified in the CAD cohort and in genome-wide association studies (GWAS).

**Results:** The results showed that RNF181 and eight other hub genes are perturbed during CAD in heart tissues. Additionally, the expression of RNF181 was validated using RT-PCR and immunohistochemistry (IHC) staining in two cardiotoxicity mouse models. The association was further verified in the CAD patient cohort and in GWAS.

**Conclusion:** Our findings illustrated for the first time that the E3 ubiquitination ligase protein RNF181 may serve as a potential biomarker in CAD, but further *in vivo* validation is warranted.

## Introduction

Coronary artery disease (CAD) has been noted as a challenge to public health faced by most industrialized and developing countries (Naghavi et al., 2017). Cardiovascular diseases (CVDs) contribute to one-third of the total deaths in the overall population (Joseph et al., 2017). According to the American Heart Association (AHA), in 2021, about 18.2 million (approx. 6.7%) adults (20 years or older) suffered from CAD; meanwhile, the threat has reached middle-aged patients, with about 2 in 10 deaths from CAD occurring in adults younger than 65 years (Virani et al., 2021). The immeasurable cost and huge burden of fatality are detrimental to the economy and society.

Vast endeavors have been put into understanding the pathology of CAD and its therapeutic strategies. For the past two decades, the discovery of novel targets, effective diagnostics, and new treatments for CAD has led to an over 50% decrease in mortality rate in the United States (Yahagi et al., 2016). CAD is a progressive cardiovascular disease that develops following atherosclerotic plaque formation or atherosclerotic occlusion of major arteries in the heart (Fuster et al., 1992). In this pathological process, fatty acid metabolism, glucose oxidation, mitochondrial fission, and oxidative stress largely impact the prognosis of the disease. Strategic treatments in clinical practice include carnitine palmitoyltransferase-1 (CPT-1) inhibitors (etomoxir, oxfenicine, etc.), malonyl-CoA inhibitors (trimetazidine), β-blockers, anti-ischemic agents, and the novel mitochondrial dynamic modulators, such as the Drp1 inhibitor. Nevertheless, these drugs have various disadvantages: inhibition of CPT-1 causes lipotoxicity in the pathological heart, leading to cardiac exacerbation; malonyl CoA inhibitor impedes the synthesis of fatty acid, but causes neuronal and cognition side effects to the brain; Drp1 is able to inhibit mitochondrial fission under oxidative stress conditions—however, its long-term effectiveness and safety remain to be discussed. For most treatments with a β-blocker or an anti-ischemic agent, they were only symptomatic; thus, further insight into identifying the risk factor for CAD is required.

The risk of CAD is a mixed consequence of genetic and lifestyle factors (Khera et al., 2016), such as smoking, physical inactivity, and a high-lipid diet, and hypertension, diabetes and obesity, or a family history (Joseph et al., 2017). In addition, the transcriptional and epigenetic regulation of macrophages and posttranslational modifications have been reported to be correlated with this complex disease (Kuznetsova et al., 2020). For example, the discovery of an abnormality in the hypermethylated region at HIF3A or the expression level of JCAD/KIAA1462 was reported to promote CAD (Xu et al., 2019). So far, the causative relationship of the perturbed genes in CAD has not been fully illustrated.

One significant contributing factor to CAD is genetic heritability. About 50% of genetic heritability was reported to be influential in the progression of CAD (Marenberg et al., 1994; Won et al., 2015). Advanced microarray and high-throughput sequencing technology have changed the research of CAD genetics. Large biobanks or shared data sources now provide huge amounts of genetic and clinical information to facilitate the discovery of risk genes. Recently, numerous genome-wide association study (GWAS) meta-analyses have identified the risk variant or mutation associated with CAD. In 2015, Nikpay et al. reported 58 susceptible loci, including rs180803 and rs12976411, that were involved in 185,000 CAD and control cases (*p* < 5 × 10^−8^) (Nikpay et al., 2015). Braenne et al. reported 159 loci, including rs1137524 and rs1060407, that may be single nucleotide polymorphisms (SNPs) with genome-wide significance in CAD (Brænne et al., 2015). The GWAS conducted by the CARDIoGRAMplusC4D Consortium reported 15 genome-wide significant loci out of 63,746 CAD cases and 130,681 controls (Deloukas et al., 2013). However, this only accounted for 10.6% of the total CAD heritability. Given that most of the locations of the loci or SNP variants identified from GWAS were outside the protein-coding regions, 40% of the risk variants were suspected to correlate with CAD, while robust associations for coding variants were only shown in four (Genetics et al., 2016). To this end, GWAS linked the associated locus or SNP to CAD pathogenesis, but further understanding of CAD heart tissue is needed to interpret the underlying mechanisms and associated genetic heredity.

Apart from GWAS, there are several other methods for the interpretation of significant genes and their associations with clinical traits. Many genetic-based prediction tools with computation scoring functions, such as the genetic risk score (GRS), gene set enrichment analysis (GSEA), differential expression analysis (DEA) and weighted gene co-expression network analysis (WGCNA), have been developed to facilitate the analysis of the genetics of CAD, either to estimate the probability of CAD or prioritize novel risk genes (Ntalla et al., 2019) based on the gene expression profiles.

WGCNA is a widely used computational method based on the gene expression profiles and clinical traits. WGCNA outperforms other analysis methods in detecting correlated gene modules. The hierarchy clustering function entails module finding, which consists of highly correlated genes, and identifies gene module–trait relationship, extracting significant genes from biologically meaningful modules (Goh et al., 2007; Horvath and Dong, 2008). WGCNA has been applied to identify modules and hub genes associated with clinical traits (Zhang and Horvath, 2005) and then to explore causal genes of diseases (Zheng et al., 2015; Wang et al., 2017; Jiao et al., 2020). WGCNA not only avoids the problems of multi-testing inherent in microarray data analysis but also provides means to bridge the gap from individual genes to systems oncology. Giulietti et al. (2018) reported the expression of two long non-coding RNAs (lncRNAs), LINC00675 and LINC01133, associated with the development and progression of pancreatic cancer using WGCNA. Zhou et al. (2018) found that hsa-miR-125b-5p, hsa-miR-145-5p, hsa-let-7c-5p, hsa-miR-218-5p, and hsa-miR-125b-2-3p were hub microRNAs (miRNAs) related to the prognosis of colon cancer. Yet, so far, the causative relationship between risk genes and CAD has not been fully illustrated.

This result further concluded the correlation between the ubiquitin–proteasome system (UPS) molecules and CAD. The significance of UPS molecules, especially E3 ligases, has not been overwhelmingly discussed. Recent studies have emphasized E3 ligases and their contribution to cardiac diseases in CVD physiology. Chen et al. (2012) illustrated that the loss of E3 ligase activity promoted the impaired protein degradation in hypertrophic cardiomyopathy. Xu et al. (2020) reported that the ubiquitin-conjugating enzyme E2 variant 1 (Ube2v1) positively regulated protein aggregation by modulating UPS in cardiomyocytes, partially by enhancing K63 ubiquitination during a proteotoxic stimulus, supporting the hypothesis that UPS-mediated proteotoxic intracellular protein aggregation and degradation may lead to the progression of cardiac disease. From this point of view, targeting the association between UPS and CAD may be important in facilitating the understanding of the underlying mechanism and causal gene identification in CAD.

In this study, WGCNA was conducted in order to identify causal genes associated with CAD. With module clustering, pathway enrichment analysis, and cardiotoxicity mouse models, one E3 ubiquitin ligase gene, *RNF181*, was identified as the causal gene for CAD in the genome, messenger RNA (mRNA) and protein levels. Furthermore, by GWAS meta-analysis, two risk variants located at the *RNF181* locus were identified as associated with coronary heart disease. Our results revealed for the first time that *RNF181* may be a causal gene for CAD, possibly through decreasing the degradation of VEGF2 mediated by the NEDD4 and ERK/MAPK signaling pathways. Thus, targeting *RNF181* might be beneficial for the treatment of CAD.

## Materials and Methods

### Microarray Data Resources and Preprocessing

The general workflow chart for this study is shown in Supplementary Figure S1. A total of 5 expression profiles from the Gene Expression Omnibus (GEO) database of NCBI, namely, GSE12504 (Ghorbel et al., 2010), GSE22253, GSE77263, GSE20681 (Beineke et al., 2012), and GSE49925 (Kim et al., 2014), were acquired, including expression profiles from CAD and control heart tissue and peripheral whole blood samples from human CAD cases. These datasets included the mRNA expression matrix, probe annotation table, and corresponding clinical features. Annotation and normalization for gene symbol and expression values were performed, respectively. The genetic profiles from the CAD cohort of the PREDICT trial (GSE20681) were collected to examine the association between causal genes in patients.

### Removal of Batch Effect

Normalization and batch effect removal were done for the combined dataset with the R package sva (Leek et al., 2012). The gene expression levels of both datasets were log2 transformed. Normalization was performed to obtain clean data by removing the background variance between samples. The proportions of males in both healthy control and CAD groups were calculated and compared with the pairwise proportion test in R. Furthermore, genes with an expression level of the lowest 25% proportion were pruned.

### Identification of DEGs

The differentially expressed genes (DEGs) between the groups of healthy controls and CAD cases were calculated with the limma package (v3.42.2) (Smyth, 2005). The *p*-value was adjusted with the Benjamini–Hochberg (BH) method, and the fold change of all genes was log2 transformed for normalization of the expression level. Moreover, the DEGs are shown in a volcano plot (Supplementary Figure S3A). A total of 1,210 DEGs with absolute log2FoldChange over 1 and *p* < 0.05 were identified.

### Construction of the Weighted Gene Co-Expression Network

A co-expression network is a widely used concept in biological interactions. It allows the interpretation of biological functionality in a system level. The conception of network construction is intuitive: some nodes (genes) are connected and co-expressed as a network across samples.

The most popular analysis pipeline for the construction of a co-expression network is WGCNA. WGCNA finds clusters of highly correlated genes (with hierarchical clustering) and summarizes these clusters by module eigengene (ME) or hub gene, in a way to liaise with external sample traits and assign module membership (MM) to genes. The branches of the hierarchical clustering dendrogram represent the modules and are refined with the dynamic tree cut method. The resulting gene clusters are often biologically meaningful. One of the advantages is that WGCNA losses less information on gene correlations and avoids the problem of multiple testing.

In WGCNA, a beta parameter is selected as a soft threshold power (SFT) to construct a co-expression network that achieves a scale-free connectivity. By adjusting the SFT, a Pearson’s correlation matrix is established for calculating the pairwise correlation matrix between genes. Then, to reach a scale-free connectivity between genes (*R* ^2^ > 0.85), the Pearson’s correlation matrix is subsequently transformed to a weighted adjacency matrix by setting a series of beta values (Ponsuksili et al., 2013). Next, a conversion of the weighted adjacency matrix to the topological overlapping matrix (TOM) is made using the block-wise module function of WGCNA. Based on the TOM, gene modules with similar expression patterns are identified with the hierarchy average lineage clustering in response to the dissimilarity of genes. For any module, module significance (MS) is defined as the Pearson’s correlation coefficient between the ME and CAD traits. The module with the highest MS is selected as the causal gene module with high association with the disease condition. Then, the relationship between the ME of the module and the CAD trait is established to reveal its significance in CAD (Langfelder and Horvath, 2014).

### Pathway Enrichment Analysis With Gene Ontology and Kyoto Encyclopedia of Gene and Genomes

For the purpose of exploring the biological function of the module and hub genes correlated with the CAD phenotype, we performed Gene Ontology (GO) (Consortium, 2015) and Kyoto Encyclopedia of Gene and Genomes (KEGG) (Kanehisa et al., 2016) analyses with the R package clusterProfiler (Yu et al., 2012). To describe gene functions, the enriched terms were assigned into three GO categories: biological process (BP), cellular component (CC), and molecular function (MF). KEGG pathway enrichment analysis was performed to determine the significant KEGG pathway terms enriched by genes (Kanehisa and Goto, 2000). Each term was calculated with a *p*-value using Fisher’s exact test, with a significance level of *p* < 0.05.

### PPI and Hub Gene Identification

In the study, the turquoise module was analyzed. Furthermore, the protein–protein interactions (PPIs) between the hub genes were queried from the STRING database and a cutoff threshold of the combined score >0.4 (as default) was set. Genes with intramodular gene significance (GS) over 0.3 and MM over 0.8 were defined as having robust correlation. For hub gene identification, overlapping genes with GO/KEGG enriched pathways or DEGs for the significant module were often considered as hub genes.

### Animals

The Animal Ethics Committee of Nankai University approved the protocol of *in vivo* studies. C57BL/6J wild-type mice were purchased from Vital River Laboratory Animal Technology Co., Ltd. (Beijing, China). These animals were placed in the animal center of Nankai University in Tianjin, China, under specific pathogen-free conditions and had free access to water and food. All animal experiments were conducted in accordance with the ARRIVE guidelines and in accordance with the National Institutes of Health Guidelines for the Care and Use of Laboratory Animals (NIH publication no. 8023, revised in 1978).

### DOX/ISO-Induced Cardiotoxicity Mouse Experiment

Eight-week-old male C57BL/6 mice were assigned into a control group and a doxycycline (DOX) group (3 per group). The following treatments were given: mice in the control group were fed with normal diet and injected with normal saline once a week, a total of 4 times; mice in the DOX group were fed with normal diet, and DOX was injected intraperitoneally at 5 mg/kg per body weight, once a week for four times in total. Four weeks after treatment initiation, mice in both groups were sacrificed and heart tissue samples were collected.

For the isoproterenol (ISO) group, 8-week-old male C57BL/6 mice in both control and ISO group (3 mice/group) received similar procedures, except for the subcutaneous (s.c.) injection with saline or ISO at 3 mg/kg per body weight daily for 18 days.

### Echocardiography and Electrocardiogram Tests

The operation procedures for the echocardiography and electrocardiogram (ECG) have been described in a previous study (Feng et al., 2021).

### Real-Time Quantitative Polymerase Chain Reaction

Heart tissue total RNA of the sacrificed mice was collected from the control, DOX, and ISO groups using the RNA extraction and purification kit as per the manufacturer’s protocol. Complementary DNA (cDNA) was synthesized by reverse transcription with the same amount of total RNA in each group (Ma et al., 2018), followed by quantitative real-time PCR (qRT-PCR) with the SYBR green PCR master mix purchased from Vazyme (Nanjing, China). The sequences of the primer templates are listed in Table 1. The expression levels of mRNAs, such as *DKK3*, *HP*, *NME7*, *OSXM*, *PIGF*, and *RNF181*, were normalized with the level of *GAPDH*.

**TABLE 1:** Primer sequences for qRT-PCR analysis

**Gene**	**Sense**	**Antisense**
*Mus DKK3*	CTC​GGG​GGT​ATT​TTG​CTG​TGT	TCC​TCC​TGA​GGG​TAG​TTG​AGA
*Mus HP*	GCT​ATG​TGG​AGC​ACT​TGG​TTC	CAC​CCA​TTG​CTT​CTC​GTC​GTT
*Mus NME7*	AGA​TTC​GCT​TTC​ATT​GCA​GAG​T	GAT​CCG​TCT​GTG​GGG​TAA​AAC
*Mus OXSM*	GGG​TTA​TGG​ACT​CTC​GGG​TGA​T	TGG​AAG​TGG​CAT​GTG​CGT​TGA​C
*Mus PIGF*	TCC​TTC​TTC​GTG​GAC​AAC​TTC​T	AGA​GGA​CAC​ATT​CGG​TTT​CAC​TA
*Mus RNF181*	TTT​GAG​GAC​CTG​GGA​TTG​GTA	TTG​GCG​CTA​CTG​ATG​ACT​GTT

### Hematoxylin–Eosin and Immunohistochemistry Staining

Heart tissues were collected from sacrificed mice of the control, DOX, and ISO groups, fixed in 4% paraformaldehyde for 24 h, and embedded in paraffin. After preparation, hematoxylin–eosin (HE) and immunohistochemistry (IHC) stainings were performed on paraffin-embedded 5-µm heart sections. The epitopes of the slices were extracted in 10 mmol/L citric acid buffer and heated at pH 7.2 in a microwave. The slides were then incubated with mouse rnf181 primary antibody overnight at 4 C and incubation performed with a horseradish peroxidase (HRP) binding secondary antibody for 1 h at room temperature. The substrate diaminophenyl guanidine (DAB) was used to detect the antibody, and the slides were counterstained with hematoxylin. Immunostained areas of the IHC stains were evaluated and positive ratios were used for statistical analysis.

### GWAS Meta-Analysis

A total of 9 GWAS results were included. Results from the large CAD population-wise studies CARDIoGRAMplusC4D (Nikpay et al., 2015; Schunkert et al., 2011; Peden et al., 2011; Deloukas et al., 2013; Nelson et al., 2017), the Framingham Heart Study 100K Project (Larson et al., 2007), Bivariate Genome-Wide Association Scan (Siewert and Voight, 2018), and the CAD data in the UK Biobank (Fall et al., 2018) were included in our study. SNP variants located near the chromosome region of *RNF181* were analyzed. The prognostic value of each SNP in CAD groups was quantified using inverse variance weighted effect size (meta-analysis method implemented in METASOFT software, http://genetics.cs.ucla.edu/meta/) (Han and Eskin, 2011).

### Statistical Analysis

Values are represented as the mean ± SEM. All assays were done in triplicate independently. Initially, all data were analyzed with the software GraphPad Prism. The workflow of the bioinformatics analyses, including WGCNA and DEGs, was performed with R version 3.6.0. The default statistics tests and cutoff values were specified in the corresponding sections in *Materials and Methods*. The statistical significance between the two groups of experimental data was assessed with the Student’s *t*-test. The linear relationship between the gene expression levels was evaluated with Pearson’s correlation coefficient. A significant difference was considered at the following levels of *p*-values: *p < 0.05; **p < 0.01; ***p < 0.001 (*n* ≥ 3).

## Results

### Defining of a List of Marker Genes Associated With CAD Implicated by GWAS and the Literature

In this study, we focused on the hub genes and the risk factors for CAD in heart tissue with in-depth insight. We began by generating a list of marker genes located within the CAD risk loci from GWAS and the literature. The keywords “coronary artery disease” and “risk gene” were searched in the literature and 158 risk gene loci were collected (Supplementary Table S1-1). As another source of risk genes, 182 independent associations with a cutoff threshold of *p* < 5.0 × 10^−8^ for CAD identified by GWAS meta-analysis were used from the 1000 Genomes Project (discovery and replication cohort with an enrolled population of ∼185,000; Supplementary Table S1-2) (Nikpay et al., 2015). For each of the 15 non-redundant lead GWAS SNPs, all RefSeq genes located within or overlapped with the region of the risk loci defined by linkage disequilibrium (LD) (*r* ^2^ ≥ 0.7) were included. The resulting CAD marker gene list contained 231 genes (Supplementary Table S1-3). Each association contained reported gene loci ranging from 1 to 16, with an average of 4.5 ± 4.7. The CAD marker gene list was enriched for GO terms such as “triglyceride homeostasis” (*p* = 1.7 × 10^−5^), “lipoprotein metabolic process” (*p* = 4.3 × 10^−7^), “vascular endothelial growth factor receptor signaling pathway” (*p* = 3.5 × 10^−5^), and “angiogenesis” (*p* = 5.7 × 10^−4^) (list shown in Supplementary Table S1-4), suggesting that it may actually contain causal genes associated with CAD.

### WGCNA Identifying Gene Modules From the Expression Profiles of Heart Tissues From CAD Patients

Firstly, a total of 50 heart tissue samples, including 20 CAD cases and 30 healthy controls from GSE12504 and GSE22253, respectively, were used to perform WGCNA. Implementation of quality controls is essential to prevent batch effects in different sequencing datasets. Thus, batch effect was removed with the package *sva*, as shown in Supplementary Figure S2. The batch effect variation analysis results showed that the sample-wise mean and *p*-value of variance were 0.472 and 0.7246, respectively. Sample-wise skewness *p*-values were also calculated, with a value of 0.3656 suggesting batch effect has been removed.

**FIGURE 1:**
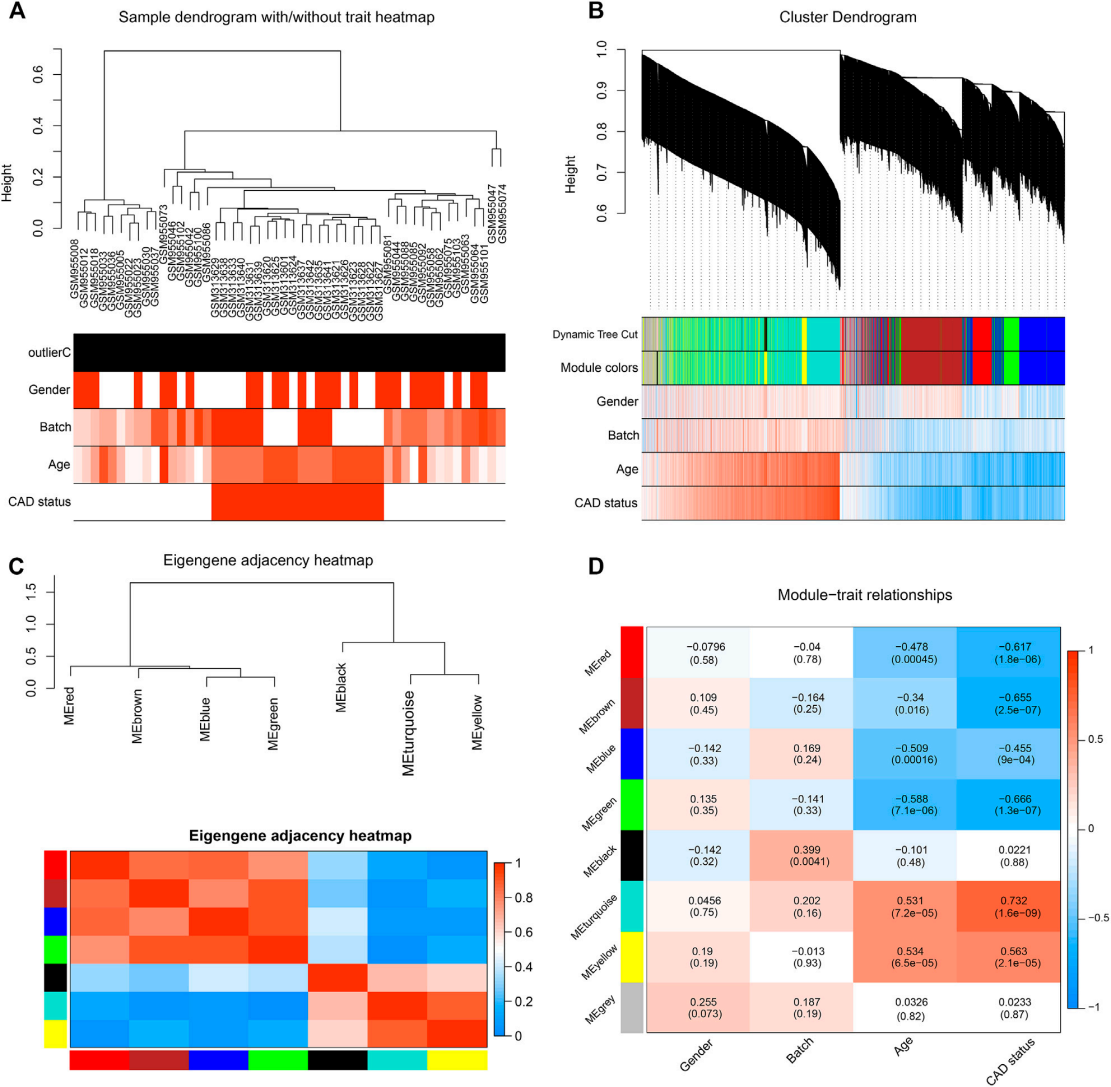
Identification of significant modules in coronary artery disease (CAD) heart tissue with weighted gene co-expression network analysis (WGCNA). **(A)** Sample dendrogram and clinical trait heatmap. Fifty samples were clustered according to clinical traits, such as gender, batch, age, and CAD status. Gender: *red* denotes male; *white*, female. Age and batch: discrete values are represented with color depth positively correlated with each sample. CAD condition: *red* represents CAD patients; *white* represents healthy controls. **(B)** Cluster dendrogram and gene–trait association heatmap obtained from the transcriptome data of GSE12504 and GSE22253 with average hierarchical linkage clustering. The *color row below the dendrogram* denotes the assigned modules allocated by dynamic tree cutting and merged module function. *Blue* and *red colors* represent a negative and a positive correlation between a gene and clinical features, respectively. **(C)** Eigengene adjacency heatmap showing extramodular connectivity among all the modules. In the heatmap, each *row* and *column* correspond to a module. *Cyan* to *blue* denotes lower module connection (<0.5); progressively *darker red* denotes higher connection (≥0.5). *Colored squares along the vertical and horizontal sides* correspond to modules. **(D)** Heatmap showing the module–trait correlation. Hierarchical clustering of eigengenes represents the modules. Each *row* denotes the module, while each *column* denotes the feature of CAD. *Values in the box* represent the correlations and corresponding *p*-values.

After data preprocessing, the expression profiles of 13,081 genes were gathered from the 50 samples. The DEGs between the CAD cases and healthy controls were analyzed with the limma package; the result showed that 1,210 DEGs were identified (Supplementary Figure S3A). Subsequently, WGCNA was performed for cluster analysis, and the quality of the dataset was evaluated with the flashClust function by sample clustering. A hierarchal clustering tree for all 50 samples in both groups was included (Figure 1A), based on which, all samples were included from the current study. With sample clustering, the 50 samples were assigned into two clusters: one containing 20 CAD samples and another containing 30 healthy controls.

After pruning low expression genes, a total of 8,525 genes in the 50 samples were included to construct the co-expression network using WGCNA. An appropriate soft thresholding power of 12 was set for the balance between the scale independence and mean connectivity between genes. Thus, a scale independence equal to 0.85 was achieved (Supplementary Figure S3B). As a result, the hierarchical clustering tree showed 7 co-expressed gene modules identified for further analysis (Figure 1B, genes not assigned to any module are shown in gray).

A TOM was built, based on which the independence among the 7 co-expression modules was analyzed. The results showed no significant overlap between module genes (Figure 1C), which suggested that a higher content of MM was achieved. The modules with the highest coefficient of correlation emerged as having the most significance in CAD. Each module was ranked by the correlation coefficient values to the CAD status.

### Measurement of Module–CAD Associations and Functional Enrichment Analysis for the Causal Gene Module

**FIGURE 2:**
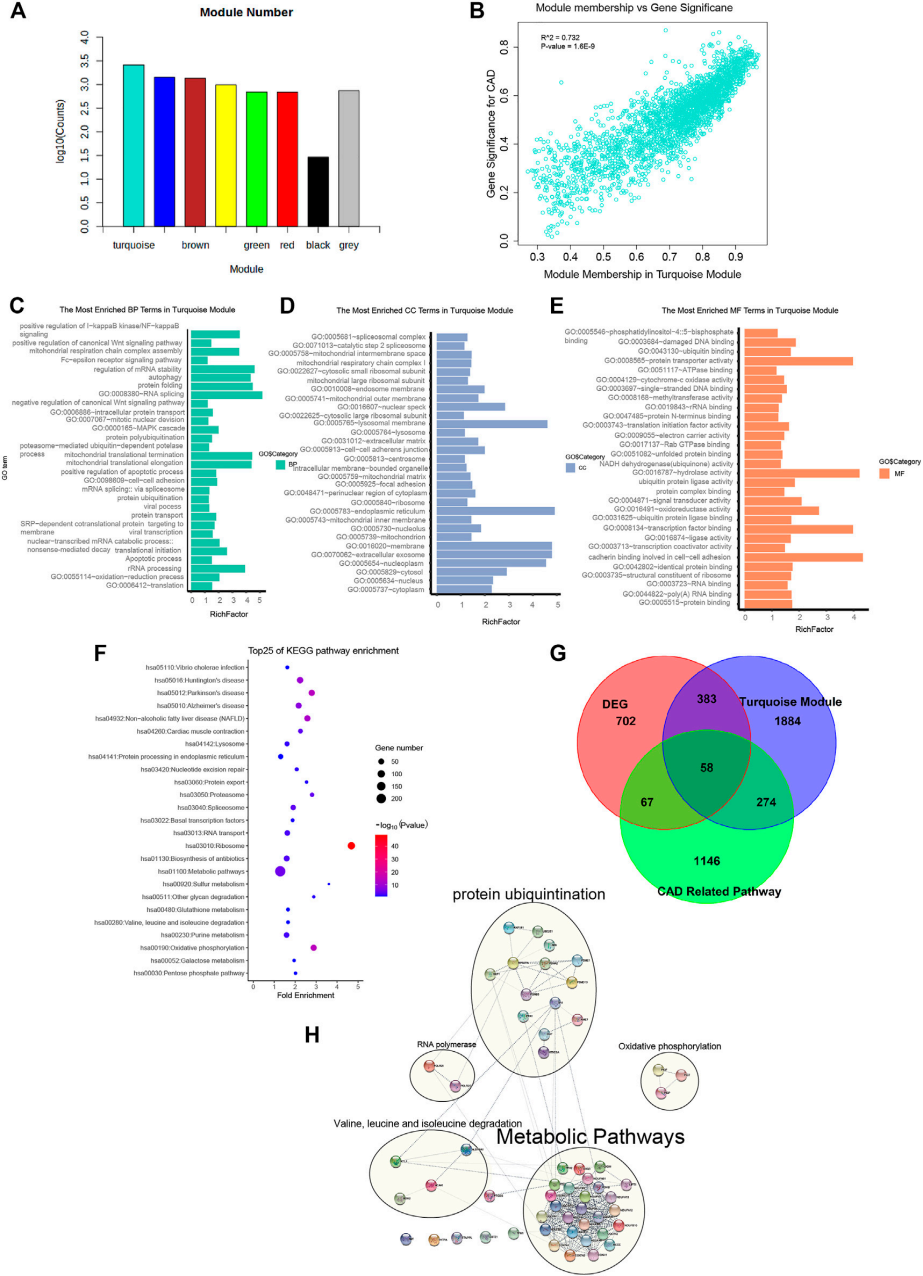
Genes from the turquoise module mainly enriched in metabolic pathways, oxidative phosphorylation, cardiac muscle contraction, and protein ubiquitination. **(A)** Gene numbers in all modules. Detailed gene symbols are listed in Supplementary Table S1. **(B)** Scatter plot showing the gene significance for coronary artery disease (CAD) *versus* module membership in the turquoise module (correlation coefficient = 0.732, *p* = 1.6e−9). **(C**–**E)** Histogram showing the Gene Ontology (GO) enrichment analysis for the genes in the turquoise module. The terms enriched in the category of molecular function (MF), biological process (BP), and cellular component (CC) are presented. The corresponding terms and adjusted *p*-values in each category are listed in Supplementary Table S1. **(F)** Bubble chart showing the enriched pathway terms referring to the Kyoto Encyclopedia of Genes and Genomes (KEGG) database for the genes of the turquoise module. **(G)** Venn diagram representing the intersection between the differentially expressed genes (DEGs), the turquoise module genes, and the genes of 4 CAD-related pathways. The intersecting 58 hub gene symbols in these three groups are listed in Supplementary Table S1. **(H)**) Subnetworks constructed by 58 CAD causal genes with protein–protein interaction. Genes were divided into 4 main subnets of biological functions based on the degree of connection.

To quantify the module–clinical feature associations, the eigengene expression dendrogram and eigengene *versus* clinical feature adjacency heatmap were constructed (Figure 1D). Seven co-expression modules with gene number ranging from 29 to 2,599 were identified (Figure 2A). Firstly, for the 7 co-expression modules, connectivity and cluster analysis was performed. The degree of association was assessed between the eigengene and CAD traits, and the corresponding GS was deemed as the correlation between each gene and traits. The arithmetical mean GS of all genes in a module was regarded as MS. For each module, the MS represents the association between its genes and CAD. The module–trait correlation heatmap showed the turquoise module as the most associated with the features of CAD, with a correlation coefficient of 0.732 (Figure 2B). Thus, the turquoise module, including a total of 2,599 genes, was identified as a causal module that positively correlated with CAD (*R* ^2^ = 0.732, *p* = 1.6E−09) (Supplementary Table S2-6).

To interpret the biological content of the genes in the turquoise module, GO and KEGG enrichment analyses were performed. In the turquoise module, 258 GO terms were enriched. The GO terms enriched in the BP category included translation (*p* = 2.80E−40), oxidation–reduction process (*p* = 9.50E−06), apoptotic process (*p* = 3.90E−02), and protein ubiquitination (*p* = 4.60E−02). Other important biological processes reported to be associated with CAD, such as MAPK cascade (*p* = 3.30E−02), Wnt signaling pathway (*p* = 2.76E−07), and NF-kB signaling pathway (*p* = 1.10E−02), were also enriched in the BP terms (Figure 2C and Supplementary Table S2-2). The GO terms of the turquoise module were enriched in the CC category of cytoplasm (*n* = 899, *p* = 9.2E−21), extracellular exosome (*n* = 524, *p* = 1.40E−17), and membrane (*n* = 384, *p* = 1.20E−08) (Figure 2D and Supplementary Table S2-3). The GO terms in the MF category were enriched in protein binding (*p* = 3E−31), cell–cell adhesion (*p* = 3.50E−03), ligase activity (*p* = 1.80E−02), and ubiquitin protein ligase binding (*p* = 9.10E−02) (Figure 2E and Supplementary Table S2-4). Referring to the KEGG database, the noted pathways enriched in genes of the turquoise module included metabolic pathways (*p* = 4.5E−6), oxidative phosphorylation (*p* = 3.7E−15), and cardiac muscle contraction (*p* = 8.0E−5) (Figure 2F and Supplementary Table S2-1).

The significance of the biological functions of the causal gene modules from CAD were implicated from the GO and KEGG analyses. The first subset was enriched in metabolic pathways such as metabolic disturbances, involving the biosynthesis and degradation of cholesterol, triglycerides, and lipoproteins, which influences the presence of CAD. The second was enriched in oxidative phosphorylation, mainly including *PIGF*, *PIGP*, and *PIGT*, which are associated with CAD due to mitigating the increased production of reactive oxygen species in the mitochondria, accumulation of mitochondrial DNA damage, and progressive respiratory chain dysfunction. The third subset was cardiac muscle contraction, impairing mitochondrial integrity predisposed by vascular cell growth in CAD. The last subset was enriched in protein ubiquitination, whereas the dysfunction of the UPS deteriorates foam cell maintenance and mitigates low-density lipoprotein (LDL) aggregation *via* mediating the ubiquitination and degradation of p53.

In summary, we identified the turquoise module as a causal module for CAD. GO and KEGG enrichment analyses revealed that the modular genes shared a high association with biological functions such as metabolic pathways, oxidative phosphorylation, cardiac muscle contraction, and protein ubiquitination.

### Identification of Hub Genes From the CAD Casual Module With DEGs and PPI

Inferred from the above-mentioned results, the turquoise gene module was identified as a casual module associated with CAD. To filter for hub genes, genes in the turquoise module were compared with 1,210 DEGs (Supplementary Table S2-5), and 441 overlapping genes were deemed as significant genes (Figure 2G and Supplementary Table S2-7). Further analysis of pathways, including metabolic pathways, oxidative phosphorylation, cardiac muscle contraction, and protein ubiquitination (a total of 1,545 genes), yielded 58 genes (Supplementary Table S2-8). Moreover, the list of 57 causal CAD genes was queried in the STRING for PPI network (Figure 2H and Supplementary Table S2-9) and to further distinguish subnetworks. Genes were divided into 4 main subnets of biological functions based on the degree of connection. Subnetwork analysis indicated that most of the genes participated in metabolic pathways, oxidative phosphorylation, cardiac muscle contraction, and protein ubiquitination.

### Validation of the Hub Genes With Gene and Protein Expressions in HMDP and HPA

To improve the performance of the gene co-expression analysis, we incorporated prior knowledge for the purpose of extracting modules with biological meanings. Thus, further validation of the expression of hub genes was performed on the mouse heart tissue samples from the Hybrid Mouse Diversity Panel (HMDP). Another analysis framework with WGCNA to discover the intrinsic differences between similar tissues (Abbassi-Daloii et al., 2020) was performed on the murine heart profiling of HMDP (GSE77263). To select an optimal set of WGCNA parameters, a total of 270 combinations of parameters, including power, minimum cluster size, deep split value, and tree cutting height, were tested for co-expression network construction. The selection of the best parameters considered prior knowledge of gene–gene interactions, including the enrichment terms of pathway databases and the minimum size of the gray module (genes not assigned with any module) (Supplementary Figure S3C). The soft thresholding power was set as 6 and the minimum cluster size as 15; the deepSplit parameter was set as 3 and the tree cutoff height as 0.15 (Supplementary Figure S3C). With the optimal parameters, WGCNA generated 57 modules, which included 7,211 genes (5,186 non-redundant gene symbols), including the gray module with a total of 675 genes. Of which, 12 significant modules were identified with a threshold of false discovery rate (FDR) < 0.05.99 of the 231 marker genes that were assigned to at least one module, suggesting that most of the CAD-related genes were enriched (Supplementary Figure S3D, 4A). Of the 58 hub genes, 35 were assigned to at least one module.

To verify the heart tissue specificity, we further investigated the 58 hub genes based on the protein and mRNA expression levels in the Human Protein Atlas (HPA). The following criteria were applied for screening CAD causal genes from the 58 genes: 1) mRNA expression was detectable in heart tissue; 2) the protein expression of the genes was reported in human heart tissues; and 3) medium to high levels of IHC staining in the left ventricle heart slices in HPA. The genes meeting these criteria included *DKK3*, *HP*, *BDH2*, *NME7*, *PIGF*, *OXSM*, *PSMD10*, *RNF181*, and *TRIM69* (Supplementary Figure S3E). Therefore, these 9 hub genes were speculated to possibly play critical roles during the pathogenesis of CAD.

### Determination of Hub Gene Perturbation in the DOX- and ISO-Induced CAD Mouse Model

To verify the expression of the hub genes derived from the WGCNA microarray in CAD heart tissue, DOX- and ISO-induced cardiotoxicity mouse models were introduced.

**FIGURE 3:**
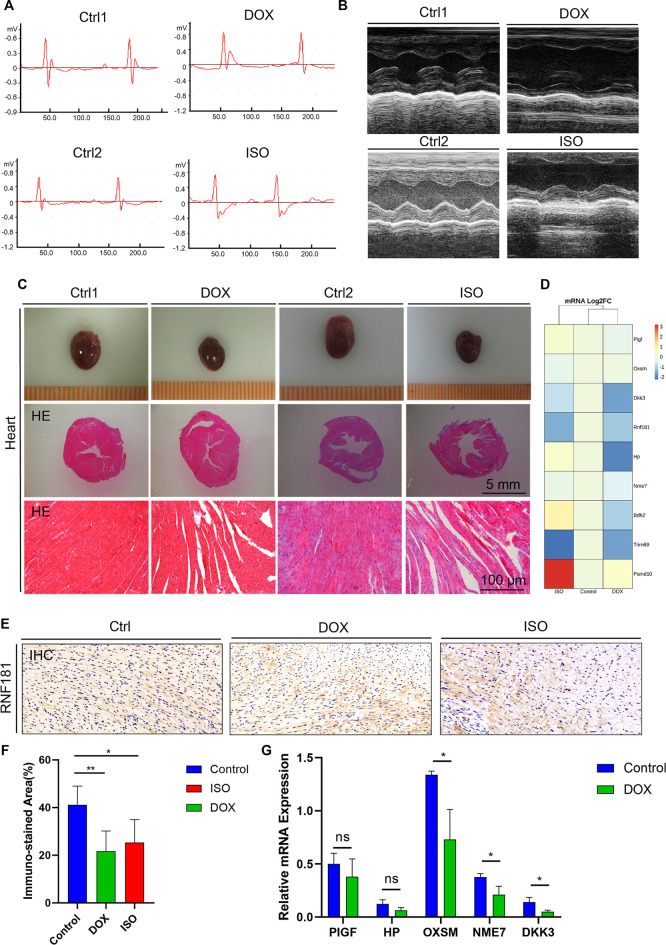
Identification and validation of the coronary artery disease (CAD) causal genes. **(A**,**B)** Diagram representing mouse ECG **(A)** and echocardiography **(B)** at the endpoint of doxycycline (DOX) or isoproterenol (ISO) treatment. **(C)** Representative pictures of the heart morphology and HE staining. **(D)** Heatmap generated from the DNA-seq expression profiles showing the Log2FoldChange of the expression of 9 CAD causal genes from the control and ISO- and DOX-treated cardiotoxicity mouse models. **(D**,**E)** Heart sections and immunohistochemistry (IHC) staining of *RNF181* in the control and DOX- and ISO-treated mice. **(F)** Statistical analysis results of the immunostained area of *RNF181* (percentage). **(G)** CAD causal genes substantially perturbed by DOX were further confirmed by qRT-PCR. Significant threshold of *p* = 0.05.

DOX and ISO are two typical agents that can cause myocardial damage by mitigating oxidative stress, necrosis, and cardiac-related metabolic disorders. To investigate the disturbance of the CAD causal genes during the myocardial damage induced by ISO or DOX, mice were treated with s.c. injection of both agents, as previously described (Feng et al., 2021). Both ECG and echocardiography tests were conducted at the end of the treatment. Compared with the control group, an increased ST segment in the ECG was observed in both DOX and ISO treatment groups (Figure 3A), which was related to the decrease of the ejection fraction (EF) and fractional shortening (FS) (Figure 3B), indicating that DOX and ISO caused heart dysfunction.

Subsequently, to determine the heart morphology affected by DOX or ISO treatment, HE staining was conducted on the heart sections. The results showed that DOX or ISO caused disordered arrangement of myocardial cells, and the myocardial structure of lysed muscle fibers was unclear (Figure 3C). Taken together, the results demonstrate that DOX or ISO can significantly induce heart atrophy and its related cardiac morphology in mice, suggesting the cardiac dysfunction in DOX- or ISO-induced heart injury.

To validate the change of the CAD causal genes in DOX- or ISO-induced heart injury, an RNA sequencing (RNA-seq) assay was performed on RNA extracted from mouse heart. The results indicated that the expression of several CAD causal genes associated with heart function were changed by DOX or ISO (Figure 3D). For instance, the gene expression levels of *RNF181* and *DKK3* were significantly decreased in both DOX- and ISO-induced cardiotoxicity models. Dickkopf-3 is a key vascular progenitor of the atherosclerotic plaque phenotype that mitigates the differentiation of fibroblasts into functional endothelial cells and is encoded by *DKK3* (Karamariti et al., 2018). Circulating Dickkopf-3 (He et al., 2016) and the fibroblast–endothelial cell transition are associated with CAD development. Besides, the expression levels of several other causal genes identified were also changed in heart function or cardiac diseases, such as *HP*, *BDH2*, *NME7*, *PIGF*, *OXSM*, *PSMD10*, and *TRIM69*. DOX decreased the expression of most hub genes; particularly, the decrease of *RNF181* expression in both DOX and ISO cardiotoxicity models followed a similar manner.

Subsequently, an IHC staining experiment for *RNF181* was conducted (Figure 3E). The *RNF181* immunostained area in both heart toxicity models showed a decrease of *RNF181* protein expression appearing in areas of CAD heart compared with the control heart tissue, suggesting that, at the protein level, the downregulation of *RNF181* may be correlated with CAD progression (Figure 3F). The effect of DOX on the expression of some CAD causal genes was further verified at the mRNA level by qRT-PCR. Consistent with the results of the RNA-seq assay, the mRNA levels of *OXSM*, *NME7*, and *DKK3* were also inhibited by DOX (Figure 3G). These results also demonstrate that *RNF181*, as well as *OXSM*, *NME7*, and *DKK3*, deteriorated during the heart failure process by mitigating multiple pathways.

Furthermore, to understand the causal relationship between *RNF181* and CAD, genes with a PPI with *RNF181* were identified in the STRING and INTACT databases. In both databases, the E3 ubiquitination ligase protein family members UBE2N, UBC, UBE2D2, UBB, UBE2D1, UBE2D3, UBA52, BCL10, UBE2E1, and RPS27A were shown to directly interact with *RNF181*. Moreover, 9 of the 10 aforementioned E3 ubiquitination proteins interacting with *RNF181* were reported to be related with heart-associated diseases (Supplementary Figure S4B).

To further verify the function of *RNF181*, the expression data from GSE143947 with selective knockdown of *RNF181* in MCF-7 cells were analyzed. The results showed that si*RNF181* induced the downregulation of CDK4, as well as MAPK and NEDD4 (Supplementary Figure S4C).

Recent pieces of evidence have shown that the E3 ubiquitin ligase Skp2/fbxl1 might regulate the proliferation of vascular smooth muscle cells (Wu et al., 2006) and cardiomyocytes (Tamamori-Adachi et al., 2004), which suggests that impaired UPS-mediated degradation may impede cardiomyocyte proliferation, while the silencing of *RNF181* reduced the cell proliferation induced by the expression of CDK4 and worsened proliferation and deteriorated cardiac efficiency post-ischemia *in vivo*.

Wang et al. reported that *RNF181* exhibits an inhibitory role in the ERK/MAPK signaling pathway, the mechanism of which was through the control of the activity of the cyclin D1–CDK4 signaling cascade. Its consequent regulatory role on the cell fate from the G1 to the S phase in gastric cancer led to a deteriorated prognosis (Wang et al., 2019). In our results, the silencing of *RNF181* was associated with the downregulation of the expressions of MAPK8 and MAPK14.

In addition, vital signaling pathways in the cardiovascular system, such as the vascular endothelial growth factor (VEGF) pathway, are also regulated by UPS. The VEGF signaling pathway mitigates angiogenesis, as well as multiple cellular activities such as cell permeability, proliferation, and survival (Pagan et al., 2013). Selective silencing of *RNF181* resulted to a significant downregulation of the expression of NEDD4, suggesting a hypothesis that the silencing of *RNF181* may increase the degradation of VEGFR2 *via* NEDD4 in CAD.

### Correlation Between *RNF181* Expression and CAD Progression in the Cohort and GWAS Studies

**FIGURE 4:**
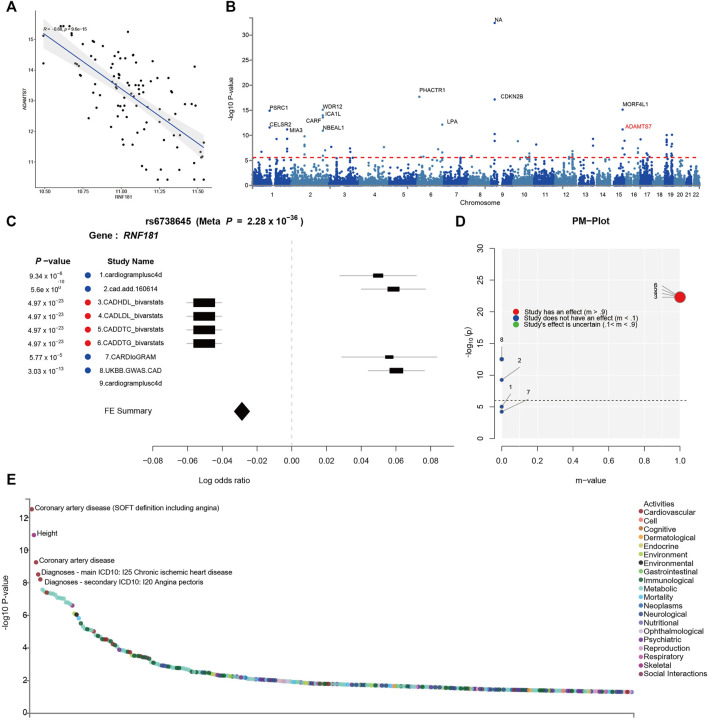
Identification of risk variants at the *RNF181* locus in the cohort and in genome-wide association studies (GWAS). The SNP locus rs6738645 in the *RNF181* chromosome region was related to coronary artery disease (CAD). **(A)** Scattered dot plot displaying Pearson’s correlation between the expression of *RNF181* and the CAD risk gene *ADAMST7* in the CAD cohort from the PREDICT trail (*R* ^2^ = 0.66, *p* < 9.6E−15). **(B)** Manhattan plot showing the top 20 SNPs in the CARDIoGRAMplusC4D GWAS results by *p*-value. **(C)** Color-coded forest plot showing the normalized effect sizes of heart tissue expression for rs6738645 and the corresponding 95% confidence intervals presented for CARDIoGRAMplusC4D and 8 other CAD meta-analyses. **(D)** *m*-values representing the posterior probability of heart tissue-specific expression and their respective *p*-values. **(E)** Dot plot of phenome-wide association studies (PheWAS) showing the expression data from GWAS ATLAS for the lead SNP (rs6738645) associated with clinical traits. Genome-wide significant expression *p*-values are shown for the indicated SNPs. *p* < 0.05 (Bonferroni corrected).

To further verify the causal relationship between *RNF181* and CAD heredity, we investigated the cohort and GWAS in the CAD population. The PREDICT trial (GSE20681) is a prospective multicenter coronary artery catheterization laboratory study conducted in the United States to identify biomarkers related to CAD (Baim et al., 2001). Pearson’s correlation was performed for the CAD cohort to investigate the expression of *RNF181* and CAD marker genes. The results showed that *RNF181* correlated with the expression levels of *ADAMTS7* and *S100A6* (Figure 4A and Supplementary Figure S5A), two well-established variant genes associated with CAD (Bauer et al., 2015; Chan et al., 2017; Cai et al., 2011; Mofid et al., 2017).

Large-scale GWAS such as the 1000 Genomes Project have identified 46 independent genome-wide significant SNPs for CAD. These SNPs included rs3918226 in *NOS3* on 7q36.1, rs10455872 and rs3798220 in *LPA* (*p* = 5.7 × 10^−39^, 4.7 × 10^−9^), and rs7412 in *APOE* (*p* = 8.2 × 10^−11^) (Nikpay et al., 2015) (Figure 4B). To identify risk variants in the *RNF181* locus related to CAD, a random-effects meta-analysis was conducted as a sensitivity analysis using the Han and Eskin method in METASOFT. To avoid significant heterogeneity, a function that mitigates power loss was utilized in the Han–Eskin method (Han and Eskin, 2011). The meta-analysis results showed that the fixed effects of gene polymorphisms on *RNF181* outcomes across CAD cohorts yielded a single SNP, rs6738645, which was therefore considered significant (with a *p*-value of 2.28 × 10^−36^). rs6738645 was considered as significantly related to CAD across the four treatment cohorts (*RNF181*: meta-analysis hazard ratio = 1.26, *p* = 3.03E−105, FDR = 1.13E−73). This effect was likely driven by the results of the Biovarstats study, where variant alleles were associated with high-density lipoprotein (HDL), LDL, and changes in total cholesterol (DTC) and triglyceride (DTG) (Figure 4C). Additionally, we also identified another association for gene variance, rs1562322, with CAD (*RNF181*: OR = 0.76, *p* = 0.290648, FDR = 0.168875) (Supplementary Figure S5B–E), suggesting that risk variants rs6738645 and rs1562322 in the *RNF181* locus may be associated with coronary heart disease.

PM analysis from the variant–trait association map of rs6738645 showed that the 4 studies had an effect on the association between rs6738645 and CAD by the *m*-value, suggesting the probability of a causal relationship (Figure 4D). Analysis of phenome-wide association studies (PheWAS) suggested that genetic variation rs6738645 is significantly associated with coronary heart disease (Pearson’s *r* = 2.437e−32, *p* = 1.19E−06) in several large GWAS. These results can be explained by the associations involving the variant rs6738645 of *RNF181*, which showed genome-wide significance in the meta-analysis for all these studies (*p* < 0.05) (Figure 4E). *RNF181* is located on chromosome 2 at chr2:85722848–85924831. In the GWAS ATLAS, the mutation of T to G at chr2:85783128 was reported in two independent studies as associated with the increase in the incidence of CAD, with enrolled populations of 148,815 and 184,305 (*p* = 3.14E−13 and 5.6E−10, respectively) (Table 2).

**TABLE 2:** Lead genome-wide association studies from GWASATLAS for the SNP rs6738645 associated with CAD clinical traits

	**PMID**	**Year**	**Domain**	**Trait**	***p*-value**	***N***	**EA**	**NEA**
3925	28714975	2017	Cardiovascular	Coronary artery disease (SOFT definition including angina)	3.14E−13	148,815	G	T
4043	30124842	2018	Skeletal	Height	1.2E−11	693,529	G	T
108	26343387	2015	Cardiovascular	Coronary artery disease	5.6E−10	184,305	G	T
3668	31427789	2019	Cardiovascular	Chronic ischemic heart disease	3.102E−09	300,791	G	T
3692	31427789	2019	Cardiovascular	Angina pectoris	6.244E−09	244,890	G	T
3470	31427789	2019	Metabolic	Trunk fat-free mass	2.666E−08	379,507	G	T
3471	31427789	2019	Metabolic	Trunk fat-free mass	3.528E−08	379,507	G	T

*EA*, effect allele; *NEA*, non-effect allele

Taken together, *RNF181* may serve as a causal gene of prognostic or therapeutic value targeting coronary heart disease.

## Discussion

The current study indicated 7 co-expression modules, one of which was significantly associated with CAD. From the causal gene module, the significance of 4 pathways, including protein ubiquitination during CAD pathogenesis, was revealed. *RNF181* and 8 other CAD causal genes were further identified from the causal gene module. The protein expression of *RNF181* was verified in both human and mouse heart toxicity samples. The gene expression of *RNF181* correlated with the expressions of ADMAST7 and S100A6 in the CAD cohort study. Further evidence in GWAS indicated that the SNP variants rs6738645 and rs1562322 in the *RNF181* locus were related to the risk of CAD. These results confirm the association between the causal gene *RNF181* and CAD progression.

Complex diseases such as CAD may be the results not only of the accumulative effects of individual genetic factors but also of gene interaction *via* biological pathway/networks. Using WGCNA on the tissue-level expression data, we found that *RNF181* was co-expressed with 7 other genes and that many of them were validated in previous studies to be associated with CAD.

Integration with the computational-based method for the discovery of novel targets in complicated diseases has been applied successfully. WGCNA and GWAS are two representative methods for identifying risk genes from genetic data. They are different in that one is based on the gene module–disease trait correlations while the other provides mutation–phenotype information from a population cohort.

A standard WGCNA workflow includes finding modules of highly correlated genes, summarizing the modules with ME or intramodular hub genes, and utilizing the eigengene network methodology to liaise modules to external traits, finally calculating MM.

One of the major shortcomings of WGCNA is that Pearson’s correlation conveys only the linear dependencies in a theoretical network. However, the true relationships observed in a biological system are sophisticated, involving both linear and nonlinear dependencies. Another is that the function of hierarchical clustering is irreversible; thus, it is impossible to readjust or relocate the uncategorized genes during module identification. Several attempts have been made in improving WGCNA. Greenfest-Allen et al. (2017) sought to improve robustness by a process of pruning uncategorized genes and performed a re-clustering step to obtain a precise module. Dai et al. (2018) introduced a modified method called cusWGCNA, which combined both the signed and unsigned network functions. Botía et al. (2017) proposed an additional *k*-means clustering step to improve the performance of WGCNA. Abbassi-Daloii et al. (2020) suggested another analysis framework with WGCNA to discover the intrinsic differences between similar tissues, which incorporated pathway knowledge and a combination of parameter selection to acquire refined gene modules with bio-meaningful content.

GWAS has been the predominant approach to the genetic analysis of complex diseases in the last decade and had demonstrated its usefulness in prioritizing over 150 novel risk loci associated with CAD. The most prominent example was *PCSK9*. Early GWAS identified that the locus rs11591147 on *PCSK9* was significantly associated with CVD (*p* = 7.5 × 10^−6^), which suggested that the Arg46Leu substitution is associated with the cholesterol levels of LDL and has favorable prognosis in cardiology. Thus far, two *PCSK9* inhibitors approved by the US Food and Drug Administration (FDA) were immediately proven as effective, which decreased the risk of cardiovascular events by decreasing ∼50% of circulating LDL cholesterol in clinical trials (Robinson et al., 2015; Sabatine et al., 2015). Thus, an integrated method of WGCNA and GWAS summary analysis was used to prioritize novel causal genes associated with CAD. The results may be suggestive in that a certain analogy could be a paradigm in prioritizing causal genes in cardiovascular diseases.

Moreover, we further discussed the association between the UPS molecule *RNF181* and CAD. As a member of the RING finger protein family, *RNF181* is a novel type of E3 ubiquitin ligase that regulates biological activities such as protein dimerization, PPI, and ubiquitin ligase activities. Thus, *RNF181* and its role in the regulation of phenotypic change, proliferation, migration, and apoptosis have been discussed in several types of malignances. *RNF181* exhibits its E3 ubiquitin ligase activity *via* binding to the integrin alpha-IIb (ITGA2B)/beta-3 (ITGB3) complex (Allen, 2015). The interaction between *RNF181* and CARD11 may enhance the NF-kB signaling pathway in lymphoma (Bedsaul et al., 2018). Besides, *RNF181* also facilitates cell viability and angiogenesis in colon carcinoma (Xiong, 2017). In breast cancer, *RNF181* prolongs the stability of *ERa* associated with AF1 *via* its RING domain binding to the domain of *ERa*, and enhancement of the gene expression of *ERa* promotes breast cancer progression (Zhu et al., 2020). In the uncurable triple-negative breast cancer (TNBC), *RNF181* inhibits the K48-linked poly-ubiquitination of YAP, thus promoting YAP stability. In this process, it mediates the activation of Hippo/YAP signaling in a positive way that prohibits the treatment of TNBC (Zhou et al., 2020). As discussed in previous studies, the UPS molecule *RNF181* played an important role in tumor biology. Our results further support the idea of *RNF181* and its association with CAD progression.

We are aware that certain limitations are present in our study. Initially, in the preprocessed CAD data, only 8,525 genes were incorporated in this study. It is likely that the coverage of related genes is incomplete and sparse, which led to only 8 positive findings with WGCNA. Certain low expressed genes that may have a causal relationship with CAD may not have been included. Also, for *RNF181* and other causal genes, bioactivity and function tests, including silencing or overexpression experiments on cardiomyocytes or assessments on druggability and pharmacology, were not performed. Even it is evolved in the DOX/ISO-induced heart toxicity model, GWAS, and the cohort study, there is no sufficient supporting evidence for clinical practice. Despite this, our results support *RNF181* being another putative novel causal gene for CAD. Subsequently, the cell origin of the RNA-seq profiles from GEO contained only tissues from the left ventricle and did not collect sorted subtypes of cells with various biological functions, including muscular cells, endothelial cells, or macrophages. Thus, we cannot immediately conclude in which specific type of cell the *RNF181*-mediated phenotype change may have functions. Lastly, WGCNA may lose power compared to the standard gene-based analysis in circumstances where the true biological mechanism is independent of gene expression.

In summary, for the first time, our findings illustrate that the E3 ubiquitination ligase *RNF181* may serve as a causal gene affecting CAD through its downregulation. *RNF181* may play an important role in CAD progression. Further efforts are required to verify the potent interactions and the regulatory mechanism of *RNF181* in CAD and other cardiovascular diseases.

## Data Availability

The datasets presented in this study can be found in online repositories. The names of the repository/repositories and accession number(s) can be found in the article/Supplementary Material.

## Supplementary Material

Supplementary Figure S1 The General Flowchart showing the analysis process of the study: Data collection, Batch effect correction, data analysis, and validation. Expression data of CAD samples from GSE12504 and normal samples from GSE22253 were analyzed after batch effect removal and preprocessing. Then, 1,210 differentially expression genes (DEGs) were screened, and the turquoise module was identified through WGCNA. GO functional and KEGG pathway enrichment analyses were performed the significant modules. CAD causal genes were identified from the module with PPI and verified with HMDP and HPA. The RNF181 and other 8 hub genes were recognized and validated using IHC, RT-PCR and gene expression in two heart toxicity mouse models. Two risk variants rs6738645 and rs1562322 at the RNF181 locus identified in GWAS meta-analysis

Supplementary Figure S2 Batch effect removal for the expression profile of CAD and healthy control heart tissue. Hierarchy clustering tree showing samples before **(A)** and after **(B)** batch effect removal with sva, each branch represents a sample, Red, CAD, and blue, healthy Control. Principle component analysis for the expression data of samples before **(C)** and after **(D)** batch effect removal. Red, CAD, blue, healthy Control. **(E, F)** Batch effect variation analysis results for the expression data of GSE12504(CAD) and GSE22253(Control).

Supplementary Figure S3 Validation of the hub genes with gene and protein expressions from HMDP and HPA. **(A)** Volcano plot showing the DEGs between CAD and Control. **(B)** Network topology analysis for adjacency matrix with a set of soft-thresholding powers. The red numbers in the drawing indicate soft-thresholding power in keeping with the correlation coefficient value. **(C)** Scattered dot plot showing the selection of parameter combinations of power, minClustersize, deepsplit and cutoff height; **(D)** Heatmap showing the gene module-traits correlation, FDR are represented as depth of color; **(E)** Cluster heatmap generated from expression profiles and clinical information of these 57 genes from the HMDP and HPA database. Gene expression (Log2FoldChange), >3, red <-2, blue. LV tissue expression, 0, white; >3.5, blue; HPA protein expression, 0, white; >3.5, purple. mRNA expression, 0, white; >3, green.

Supplementary Figure S4 siRNF181 is closely related to multiple biological processes in CAD. RNF181 PPI profiles from **(A)** STRING and **(B)** INTACT network. **(C)** Chord diagram constructed from siRNF181 data on the correlation between RNF181 downregulation and some cardiovascular process mediators or UPS molecules. Red denotes positive correlations, while the green, negative.

Supplementary Figure S5 Identification of CAD-associated risk variants rs1562322 at RNF181 locus in cohort and GWAS studies. **(A)** Scattered dot plot displaying the Pearson’s correlation between the genes expression of RNF181 and the CAD-causal gene S100A6 in the CAD cohort from the CAD cohort of predict trail. N=99, R^2^ = 0.78, p-value < 2.2E-16; **(B)** Identification of lead SNP rs1562322 in the CARDIoGRAMplusC4D and other 9 meta-analysis. **(C)** Color-coded forest plot showing the heart-tissue expression normalized effect sizes for rs1562322 and corresponding 95% confidence intervals are presented for CARDIoGRAMplusC4D and other 8 CAD meta-analysis. **(D)** m-values representing the posterior probability of heart tissue-specific expression and their respective expression P-values are also shown. **(E)** PheWAS showing the expression data from GWASATLAS for the lead GWA studies SNP rs1562322 associated with clinical traits. Genome-wide significant expression P-values are shown for indicated SNPs. P-value,Bonferroni corrected < 0.05.
