# Supplementary figures and images for: Weighted Co-Expression Network Analysis Identifies RNF181 as a Causal Gene of Coronary Artery Disease
Source: Front Genet. 2022 Feb 10;12:818813. doi: 10.3389/fgene.2021.818813 (PMC8867041; doi:10.3389/fgene.2021.818813)

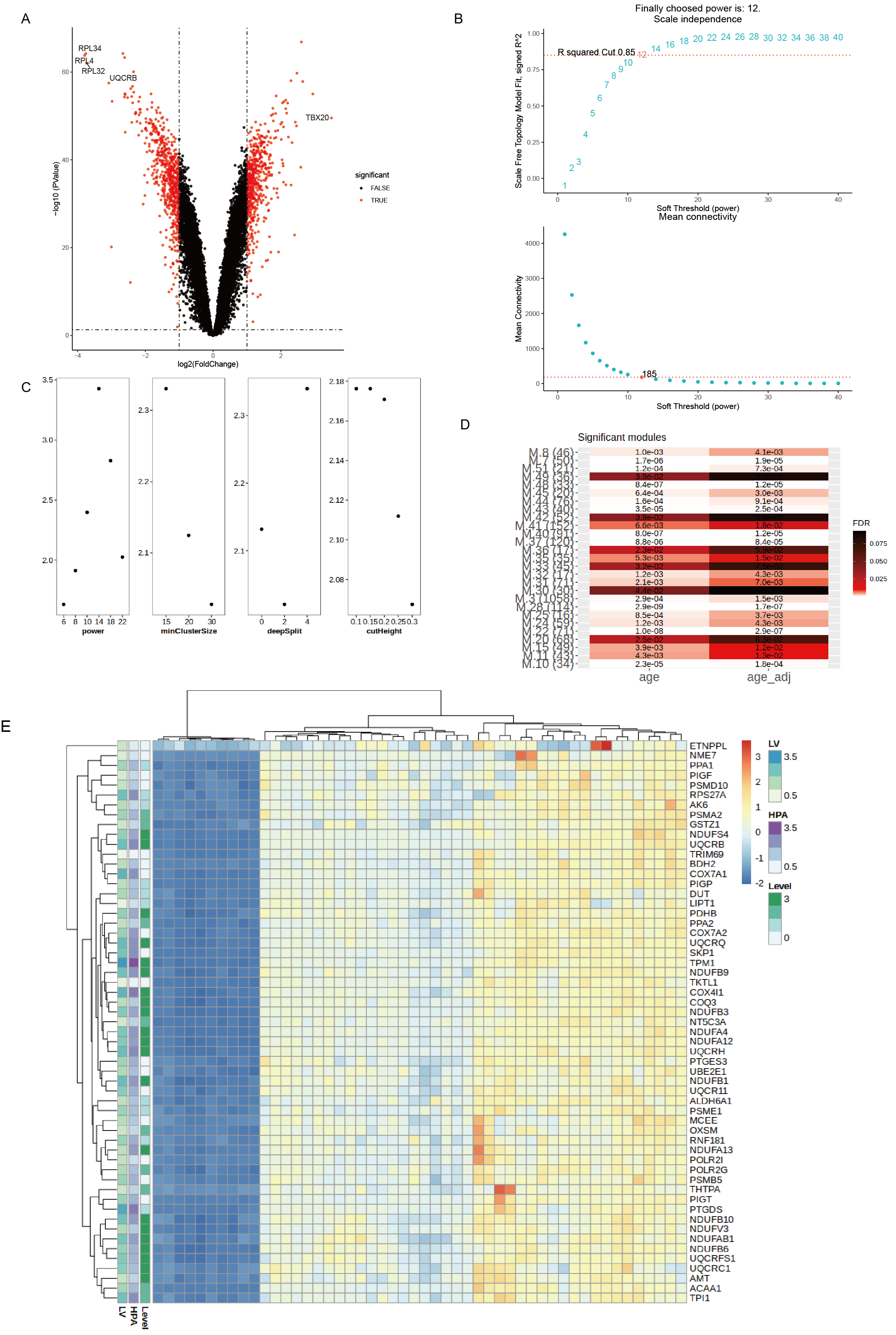

Supplement: Supplementary file 2 [file Image3.JPEG]

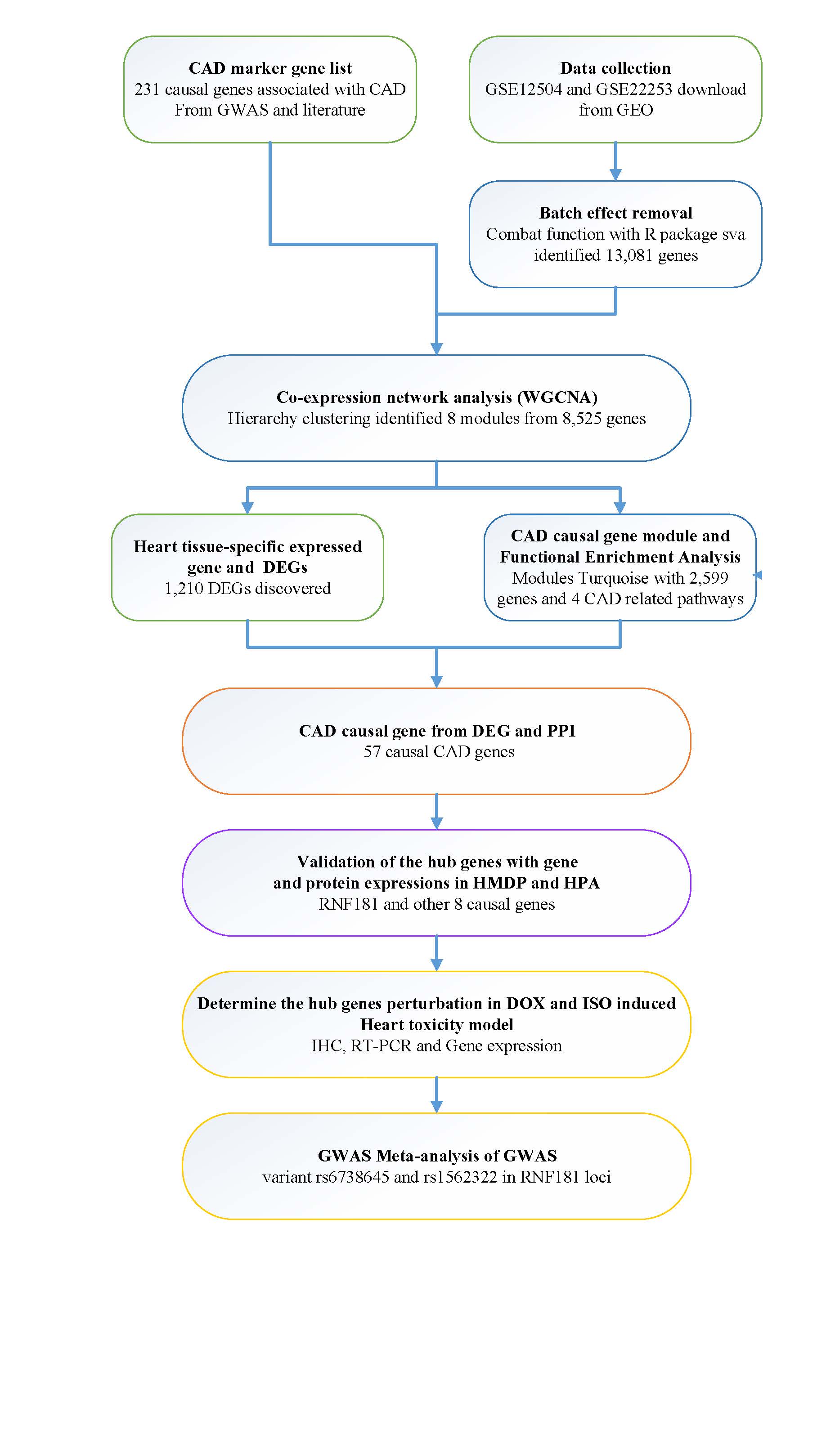

Supplement: Supplementary file 3 [file Image1.JPEG]

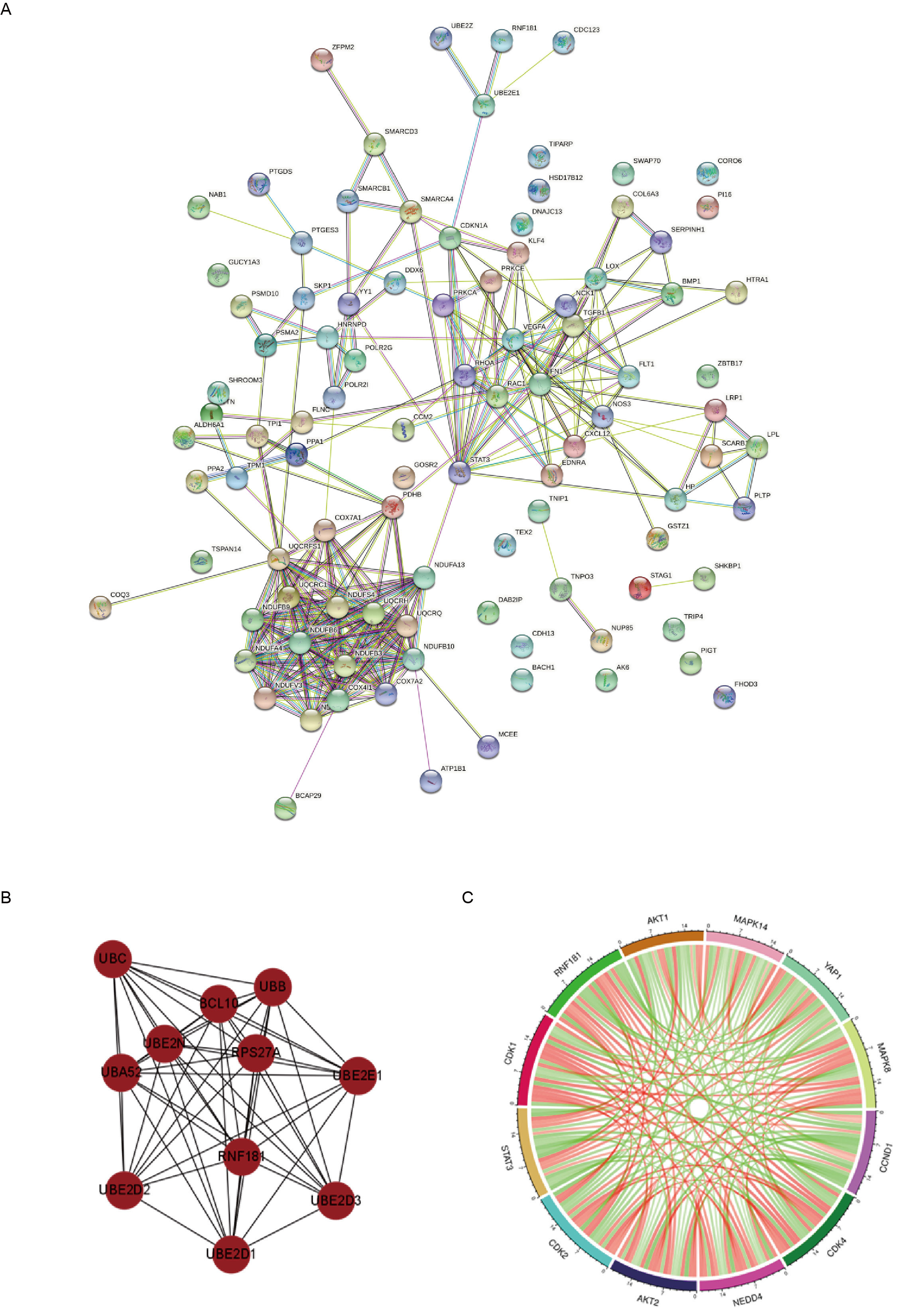

Supplement: Supplementary file 4 [file Image4.JPEG]

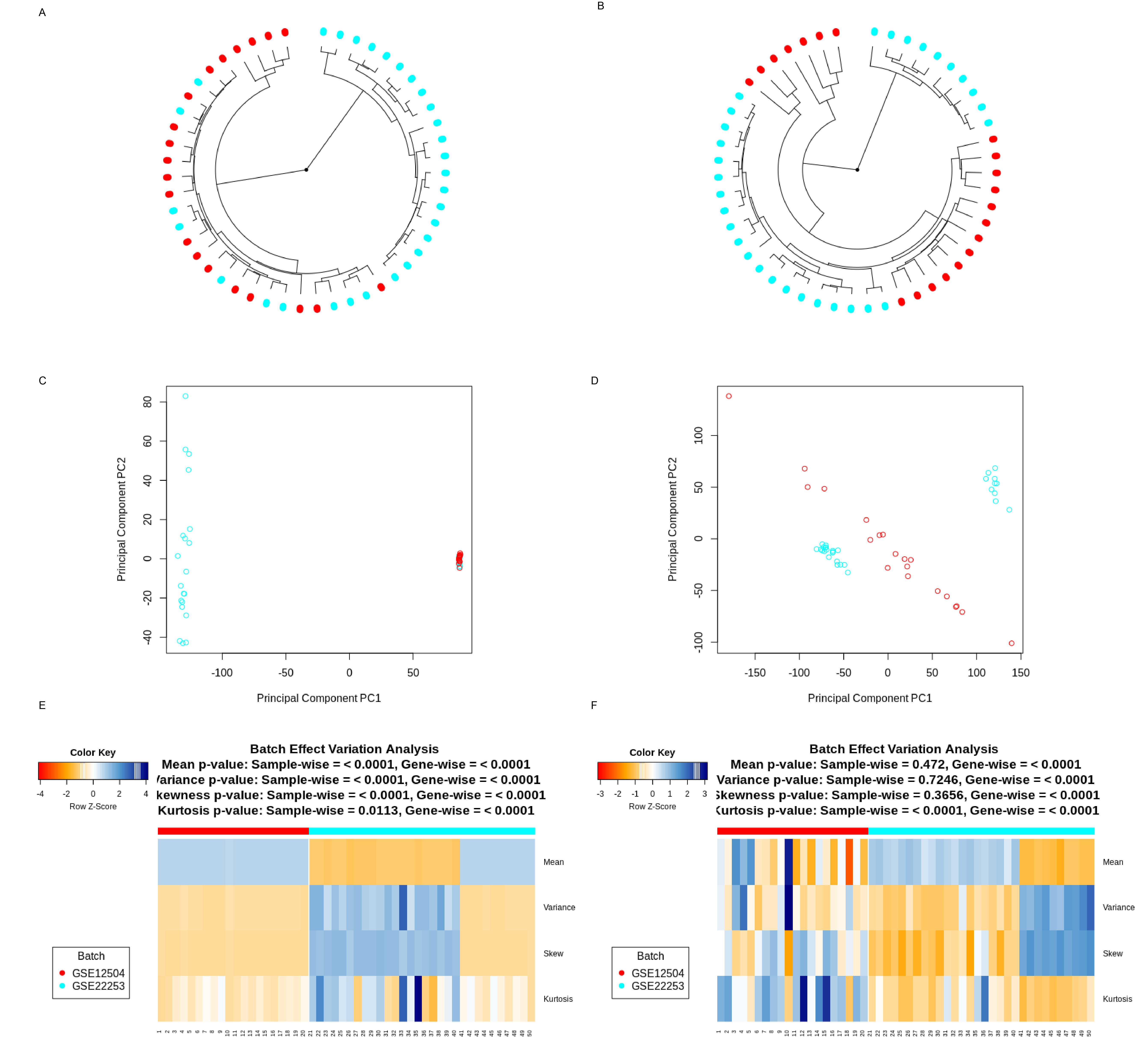

Supplement: Supplementary file 5 [file Image2.JPEG]

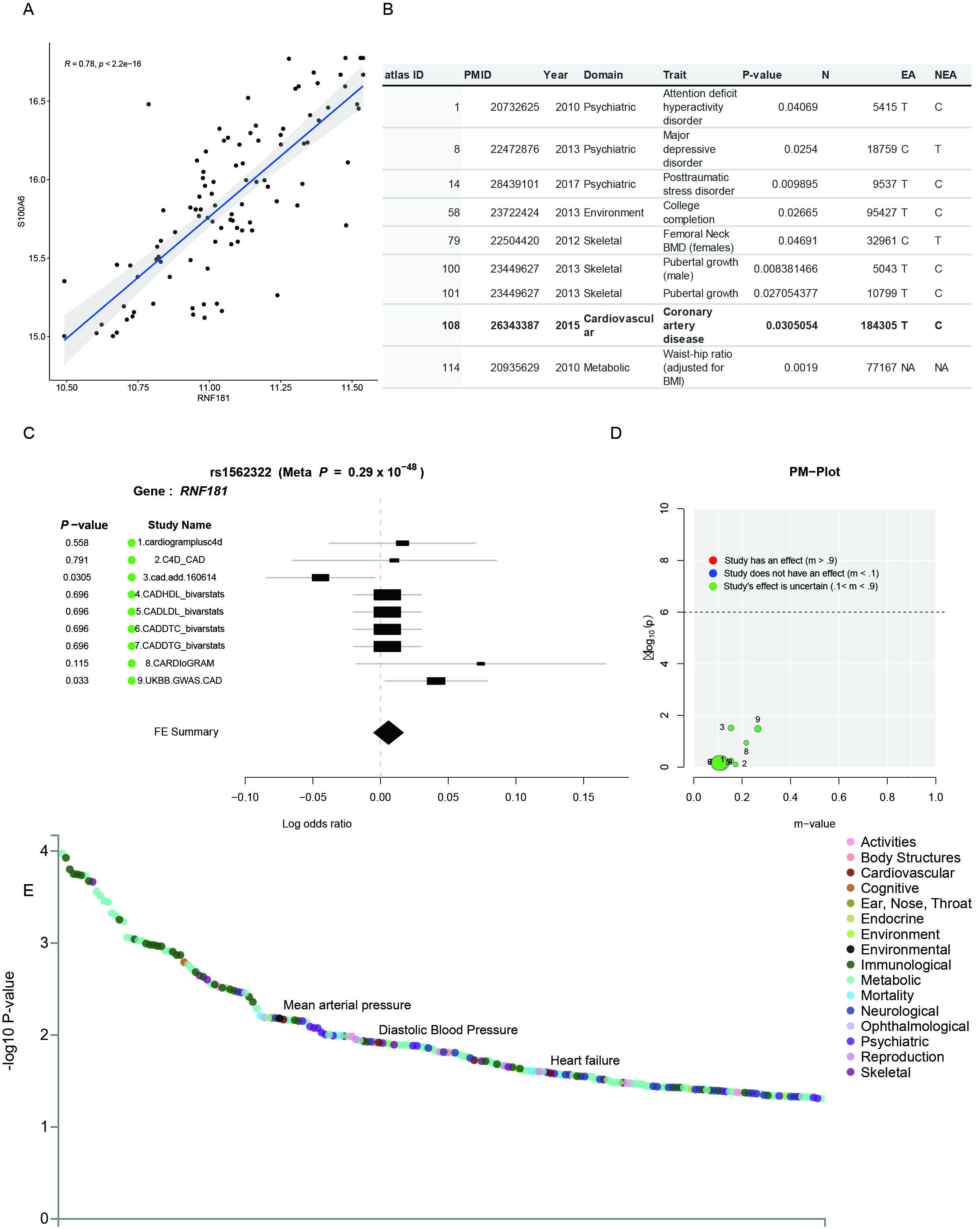

Supplement: Supplementary file 6 [file Image5.JPEG]
