# Supplementary material for: Weighted Co-Expression Network Analysis Identifies RNF181 as a Causal Gene of Coronary Artery Disease
Source: Front Genet. 2022 Feb 10;12:818813. doi: 10.3389/fgene.2021.818813 (PMC8867041; doi:10.3389/fgene.2021.818813)
Supplement: Supplementary file 7 [file Table3.DOCX]

Please be noted the original data, R code scripts, lab test data and results are uploaded to jianguoyun, with an access link: https://www.jianguoyun.com/#/sandbox/141782a/1f0e520d67297985/%2F/.
